# Acute Bilateral Vocal Cord Paralysis in a Patient With Anti-Leucine-Rich Glioma-Inactivated 1 (LGI1) Limbic Encephalitis

**DOI:** 10.7759/cureus.75475

**Published:** 2024-12-10

**Authors:** Joseph L Kim, Yohan Kim, Andres Saenz

**Affiliations:** 1 Neurology, UT Health San Antonio, San Antonio, USA

**Keywords:** anti-lgi1 le, anti-lgi1 limbic encephalitis, autoimmune neurologic disorder, lgi-1, lgi1 antibody autoimmune encephalitis, limbic encephalitis, stridor, vocal cord dysfunction, vocal cord failure, vocal cord paralysis

## Abstract

Autoimmune encephalitis is a disorder characterized by an autoantibody-mediated process that leads to brain inflammation. It is associated with neurological symptoms including cognitive issues, psychiatric problems, seizures, and autonomic dysfunctions. Anti-leucine-rich glioma-inactivated 1 limbic encephalitis (anti-LGI1 LE) is a rare type of autoimmune LE with a unique presentation, comprising neuropsychiatric disturbances, sleep disorders, and faciobrachial dystonic seizures (FBDS). While the involvement of the larynx is seen in various other autoimmune and neurological diseases, it is not a known issue with anti-LGI1 LE. The purpose of this case report is to highlight an atypical symptom - acute bilateral vocal cord failure - that unexpectedly occurred in a patient with anti-LGI1 LE.

## Introduction

Anti-leucine-rich glioma-inactivated 1 limbic encephalitis (anti-LGI1 LE) is a type of autoimmune encephalitis that is distinctly characterized by neuropsychiatric changes, sleeping disturbances, and faciobrachial dystonic seizures (FBDS). Similar to other autoimmune encephalitides, it is associated with other neurological manifestations such as encephalopathy, seizures, dysautonomia, and focal neurological deficits [1]. Despite the vast and non-specific array of symptoms, there is a less clear connection between anti-LGI1 LE and laryngeal involvement.

The larynx plays an important immunological role as an intersection between the respiratory and gastrointestinal tract [2]. It has been well documented in the literature that various autoimmune, inflammatory, paraneoplastic, and neurological conditions can cause a degree of vocal cord failure and have other effects on the larynx and bulbar system. Conditions such as myasthenia gravis, multiple sclerosis, systemic lupus erythematosus, sarcoidosis, Sjogren’s syndrome, and rheumatoid arthritis are a handful of diseases that can have some sort of laryngeal manifestations [3]. Anti-N-methyl-D-aspartate (NMDA) receptor encephalitis and anti-IgLON5 encephalitis are specific autoimmune encephalitides that have been documented to involve the vocal cords, manifesting complete paralysis, palsy, or a degree of dysphonia [4,5].

The vocal cords are made of the thyroarytenoid muscle and overlying mucosa in the larynx, and they assist with vocalization, breathing, and swallowing. The muscles are innervated by the superior laryngeal nerve and recurrent laryngeal nerve, which both branch from the vagus nerve. Vocal cord immobility refers to the loss of vocal cord function due to any etiology. When the recurrent laryngeal nerve is damaged, vocal cord paralysis occurs. Symptoms such as hoarse voice, stridor, respiratory distress, and dysphagia occur due to the inability of the vocal cords to contract and fully close [6]. The vocal cords can also be affected by immune complex depositions, lesions, nodules, and inflammation of the cartilage. Depending on the severity of the symptoms, an emergent otolaryngology evaluation may be advised to help with diagnosis and management. Serious complications such as aspiration pneumonia or respiratory failure can arise if these patients are not treated promptly [7].

We discuss a case of a male patient admitted to the hospital who developed sudden-onset stridor while being treated with plasma exchange for anti-LGI1 LE. He was found to have acute bilateral vocal cord paralysis and underwent emergent tracheostomy. This complication was attributed to his autoimmune disease as the patient had no prior medical comorbidity, risk factor, or any inciting event that could otherwise explain the vocal cord failure. This is a unique presentation in that although laryngeal involvement is seen in autoimmune encephalitides and neurological diseases as mentioned previously, vocal cord failure secondary to anti-LGI1 LE has yet to be documented.

## Case presentation

A 62-year-old male with a known history of hypertension, type 2 diabetes, seizures, and prior psychiatric hospitalizations for suicidal ideation and bizarre hallucinations, was brought in by his family to the emergency room for new-onset suicidal ideation and behavioral changes. During the past month, the patient had been experiencing progressive symptoms including inability to sleep, irritability, episodes of disorientation, speech impairment, and flattened affect. The patient had been previously compliant with his seizure and psychiatric medications, which included levetiracetam, lacosamide, aripiprazole, and bupropion.

Leading up to his current presentation, the family had noticed that the patient was not acting like his typical self. He would have episodes of confusion and spells of facial and arm “spasms” that would resolve on its own. These symptoms had persisted, and the family had decided to bring him to the emergency room after he had started endorsing suicidal thoughts and ideation similar to his previous psychiatric hospitalization episode. His initial evaluation in the emergency room showed stable vital signs and labs including a negative urine drug screening. There was no history of alcohol, tobacco, or illicit drug use, and the family denied recent illness, travel, or injuries. While waiting in the emergency room, the patient had two witnessed generalized tonic-clonic seizures, and he was evaluated and admitted to the neurology service for further care. An initial CT of the head showed no concerns related to acute bleeds. His initial EEG showed right frontal temporal seizures. Valproic acid was initiated, in addition to the existing levetiracetam and lacosamide dose.

While admitted, the patient developed various autonomic imbalance issues including hypo and hyperthermia, tachycardia, and hypotension, which were all medically and symptomatically managed. Meanwhile, the patient’s mental status continued to deteriorate, and the family was concerned with the ongoing encephalopathy. He also started to develop episodes of facial and upper extremity dystonia and “spasms” similar to those that occurred before hospitalization. These events correlated with the EEG findings and were reported to indicate FBDS. Phenytoin was then added in a stepwise manner once his other seizure medications were optimized. During the up-titration of his anti-seizure medications, a lumbar puncture was performed due to intractable seizures and continued encephalopathy. An MRI with and without contrast of his brain was subsequently performed. Finally, given his history of diabetes, high-dose steroids were deferred, and due to the progressing symptoms, the patient started a five-course treatment of plasma exchange.

**Figure 1:**
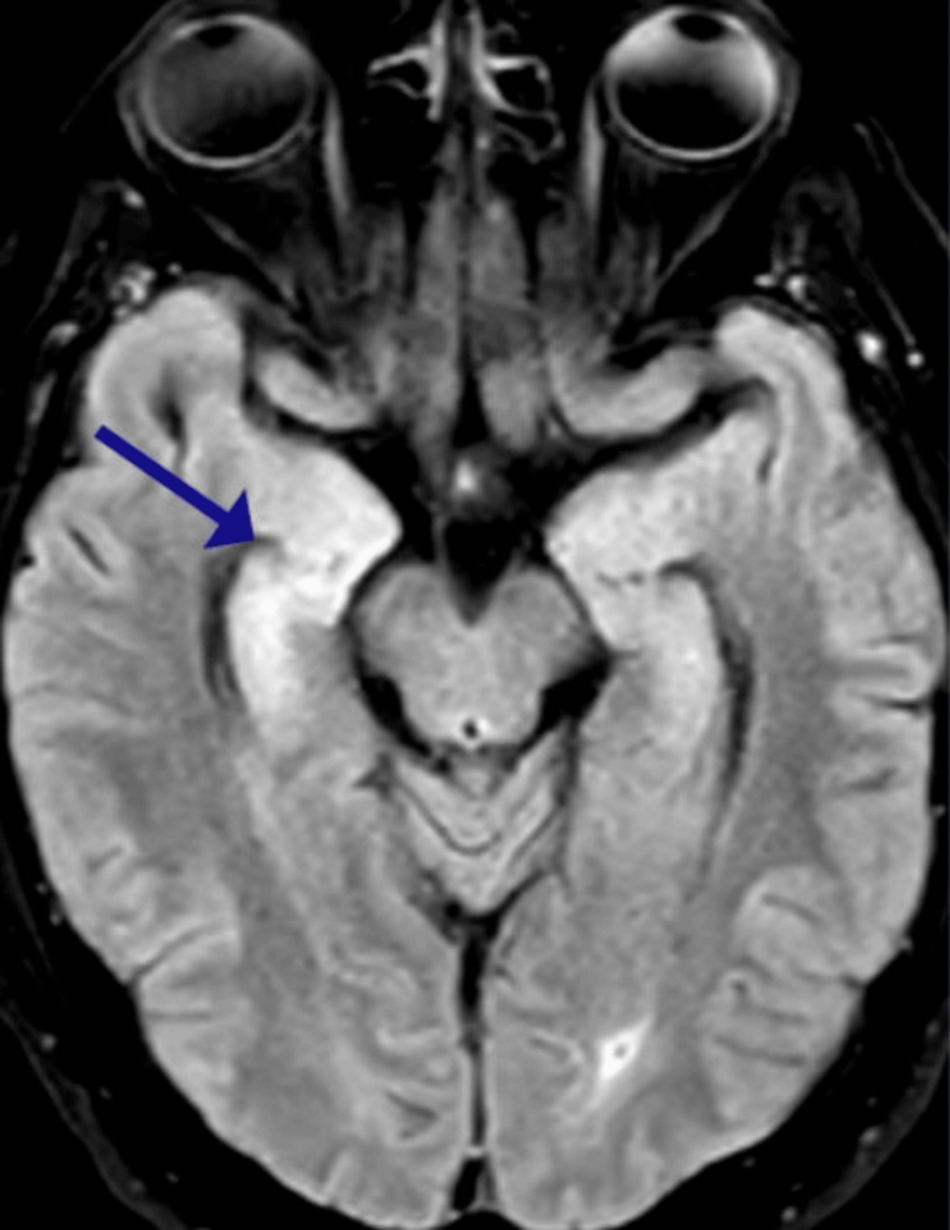
Brain MRI - axial T2 FLAIR sequence Bilateral, right (blue arrow) greater than left, T2 FLAIR hyperintensity seen on mesial temporal lobe and hippocampus FLAIR, fluid-attenuated inversion recovery; MRI, magnetic resonance imaging

The MRI brain with and without contrast revealed bilateral, asymmetric, right-greater-than-left hyperintensity of the mesial temporal lobe and hippocampus on the T2 FLAIR sequence (Figure 1). There was neither any diffusion restriction on the DWI sequence nor contrast enhancement. The cerebral spinal fluid (CSF) was sent for a broad workup. The initial CSF results showed a protein level of 29, glucose of 100, total nucleated cells less than 5, and negative bacterial and viral growth. Various other studies including RT-QuIC, paraneoplastic, and meningitis panel returned negative. Eventually, the autoimmune encephalitis panel showed positivity to anti-LGI1 IgG antibodies, and a diagnosis of anti-LGI1 LE was established.

As the patient continued to undergo treatment with plasma exchange, he started to recover from his initial presenting symptoms. His sleep improved, and the family reported that the patient was returning to his baseline behavior. In addition, the clinical frequency and intensity of the patient’s FBDS had dramatically decreased, and the EEG reports corresponded to this improvement.

The night before the fourth plasma exchange session, the patient developed increased work of breathing with audible noisy breath sounds. This worsened throughout the day, leading to a hypoxic event requiring 3 liters of oxygen via nasal cannula. A broad workup was performed. The patient had an acute leukocytosis (white blood cell count of 13.37 from 7.41), and a CT chest without contrast showed ground glass and consolidative opacities in bilateral lower lobes of the lung, raising concerns for aspiration with possible superimposed infection. The patient had a negative viral respiratory panel, and his blood and sputum cultures later returned negative. Despite starting on broad-spectrum antibiotics (vancomycin and piperacillin/tazobactam), he continued to have abnormal labored breathing. On the second day, he developed a distinct audible high-pitched breathing sound that was concerning for stridor. He was evaluated by the otorhinolaryngology service and underwent a trans-nasal flexible fiberoptic laryngoscopy procedure. He was found to have bilateral vocal cord paralysis and was emergently taken for an awake tracheostomy.

After the procedure, the patient was admitted to the surgical ICU, where he completed five days of ceftriaxone and the rest of his plasma exchange sessions (totaling five treatments). The patient recovered well and was weaned off the ventilator. Subsequent evaluation by the otorhinolaryngology service did not reveal a specific inciting event for the patient’s acute vocal cord failure, and, ultimately, both the primary neurology and otorhinolaryngology team attributed it to the patient’s anti-LGI1 LE. The patient received one dose of rituximab before being discharged and continued the rituximab infusion treatment as part of immunotherapy thereafter. On subsequent follow-up in the neurology outpatient clinic, he was stable with no flare-ups and no further incidences of seizures. His anti-seizure medications were slowly scaled back to levetiracetam and lacosamide.

## Discussion

This report describes a patient with a history of seizures and psychiatric symptoms, who, while hospitalized, experienced an acute onset of a previously unreported and novel clinical manifestation linked to anti-LGI1 LE. He was admitted to the neurology service for encephalopathy, sleep disturbances, and seizures. A brain MRI, showing bilateral mesial temporal and hippocampal lesions, and CSF studies, which were positive for anti-LGI1 IgG antibodies, led to a diagnosis of anti-LGI1 LE. After the initiation of plasma exchange, he started showing clinical improvement. While receiving plasmapheresis, he developed acute-onset stridor and was diagnosed with bilateral vocal cord paralysis following a trans-nasal flexible fiberoptic laryngoscopy procedure. He underwent an emergent awake tracheostomy and was eventually weaned off the ventilator after completing his plasma exchange treatment. He was started on rituximab infusion for his immunotherapy treatment with his seizure regimen optimized to levetiracetam and lacosamide. Given the absence of an inciting event or identifiable risk factor, his bilateral vocal cord paralysis was attributed to anti-LGI1 LE.

Anti-LGI1 LE is characterized by symptoms including neuropsychiatric disturbances, memory deterioration, epileptic seizures, sleep changes, FBDS, dysautonomia, and hyponatremia. Since other diseases can present with similar features, a comprehensive workup is still warranted, including a comprehensive blood and CSF analysis and central nervous system imaging. Treatment for the acute phase entails a short course of high-dose steroids, plasmapheresis, or intravenous immunoglobulin, with long-term immunosuppressants and anti-seizure medications prescribed for the chronic phase [1].

Vocal cord paralysis refers to the inability of one or both vocal cords to contract and close. This restriction in movement can be asymptomatic or cause dysphonia, dysphagia, or aspiration. In some severe cases, there may be audible stridor or snoring [8]. The vocal cords, which make up the larynx, are muscles that are controlled by the vagus nerve and its branches (the recurrent laryngeal nerve and superior laryngeal nerve). Damage to these nerves from trauma, surgery, or inflammation can cause either unilateral or bilateral vocal cord paralysis, with bilateral vocal cord paralysis associated with a risk of life-threatening complications [9,10].

The reason the larynx is particularly susceptible to autoimmune conditions is due to its intricate innervation, vascular supply, and mucosal microenvironment [2,11]. Because the larynx is innervated by the vagus nerve and its branches, it is highly susceptible to autoimmune conditions that cause inflammatory neuropathies [12]. Another reason pertains to the rich vascular supply of the larynx and its proximity to the lymphatic drainage system creating a greater risk of exposure to circulating autoantibodies and inflammatory markers [13]. Finally, the laryngeal mucosa houses an array of local immune cells that amplify inflammation in the area. In various conditions, it can cause dysregulation in these immune cells and create pro-inflammatory cytokines that contribute to tissue damage and inflammation [2].

Various autoimmune and neurological conditions can cause vocal cord paralysis through different immune-mediated pathways [3]. Myasthenia gravis can cause vocal cord paresis or paralysis from impaired neuromuscular transmission secondary to antibodies against acetylcholine receptors [14]. Multiple sclerosis can cause demyelinating lesions in the brainstem or cervical pathways that can affect vocal cord movement [15]. Systemic lupus erythematosus can cause vasculitis that affects the recurrent laryngeal nerve with additional mucosal ulceration and edema which can lead to vocal cord problems [16]. Finally, rheumatoid arthritis can also cause compressive neuropathy on the recurrent laryngeal nerve that can lead to vocal cord paralysis [17]. Overall, the larynx is a complex anatomical structure that is highly susceptible to various pathological conditions.

It is unclear if anti-LGI1 LE has any effect on the vocal cords, unlike other autoimmune encephalitides. It has been well-documented that anti-NMDA receptor encephalitis and anti-IgLON5 encephalitis have some laryngeal involvement [4,5]. It is thought that there is a neuroinflammatory component that affects the brainstem or peripheral nervous system contributing to a dysfunctional vocal cord. NMDA receptors play a crucial role in synaptic plasticity and neuronal communication. When there is dysfunction in the receptors, as seen in NMDA receptor encephalitis, it leads to impairment in vocal cord movement [18]. On the other hand, IgLON5 plays a role in cell adhesion and signaling. When this is affected, the motor control of the laryngeal muscle is disrupted [19]. In both instances, there is a neuroinflammatory disruption in the neural circuits, which causes vocal cord paralysis leading to dysphonia, dysphagia, and, in critical cases, airway obstruction.

This case report further demonstrates that anti-LGI1 LE also affects the vocal cords, just like other autoimmune encephalitides. LGI1 protein plays a critical role in regulating neuronal excitability in the central nervous system and is found in high concentrations in the hippocampus. In anti-LGI1 LE, there is a disruption in synaptic organization and transmission, which theoretically may be the reason for vocal cord impairment [20]. Further studies need to be conducted to clarify the exact mechanism of the role of anti-LGI1 LE and its involvement with the larynx.

## Conclusions

This report described a patient with classic symptoms of anti-LGI1 LE who was admitted to the hospital for plasma exchange and developed a symptom not previously associated with the disease. Though his problem went undiagnosed for years, he continued to have waxing and waning symptoms, eventually manifesting a new symptom as his disease progressed. Specifically, he had an acute-onset bilateral vocal cord paralysis, necessitating an emergent tracheostomy. Although there are no documented cases of vocal cord failure linked to anti-LGI1 LE, given that laryngeal involvement is common in other autoimmune encephalitides and neurological diseases and the lack of any other precipitating cause, the bilateral vocal cord paralysis was attributed to the patient’s anti-LGI1 LE. This report sheds light on anti-LGI1 LE, as several unusual presentations and symptoms are linked with this condition. Clinicians should maintain a broad differential and strive to promptly recognize this disease to enable timely and effective therapy. This report provides deeper insights into the impact of anti-LGI1 LE on the larynx, specifically bilateral vocal cord paralysis. Anti-LGI1 LE can be a clinically challenging condition and clinicians should be well-versed about its associated symptoms.
